# Development of Magnetically Active Scaffolds for Bone Regeneration

**DOI:** 10.3390/nano8090678

**Published:** 2018-08-30

**Authors:** Esperanza Díaz, Mᵃ Blanca Valle, Sylvie Ribeiro, Senentxu Lanceros-Mendez, José Manuel Barandiarán

**Affiliations:** 1 Escuela de Ingeniería de Bilbao, Departamento de Ingeniería Minera, Metalúrgica y Ciencia de Materiales, Universidad del País Vasco (UPV/EHU), 48920 Portugalete, Spain; 2 BCMaterials, Basque Centre for Materials, Applications and Nanostructures, UPV/EHU Science Park, 48940 Leioa, Spain; slm@bcmaterials.es (S.L.-M.); jm.barandiaran@ehu.eus (J.M.B.); 3 Facultad de Ciencia y Tecnología, Departamento de Electricidad y Electrónica, University of the Basque Country (UPV/EHU), Sarriena s/n, 48940 Leioa, Spain; mb.valle@ehu.eus; 4 Centro/Departamento de Física, Universidade do Minho, 4710-057 Braga, Portugal; s.ribeiro@bcmaterials.es; 5 Centre of Molecular and Environmental Biology (CBMA), Universidade do Minho, Campus de Gualtar, 4710-057 Braga, Portugal; 6 IKERBASQUE, Basque Foundation for Science, 48013 Bilbao, Spain

**Keywords:** Fe-doped hydroxyapatite, poly (l-lactide), scaffolds, magnetism, cytotoxicity

## Abstract

This work reports on the synthesis, with the thermally induced phase separation (TIPS) technique, of poly (l-lactide) (PLLA) scaffolds containing Fe-doped hydroxyapatite (FeHA) particles for bone regeneration. Magnetization curves and X-ray diffraction indicate two magnetic particle phases: FeHA and magnetite Fe_3_O_4_. Magnetic nanoparticles (MNPs) are approximately 30 ± 5 nm in width and 125 ± 25 nm in length, and show typical ferromagnetic properties, including coercivity and rapid saturation magnetization. Scanning electron microscopy (SEM) images of the magnetic scaffolds reveal their complex morphology changes with MNP concentration. Similarly, at compositions of approximately 20% MNPs, the phase separation changes, passing from solid–liquid to liquid–liquid as revealed by the hill-like structures, with low peaks that give the walls in the SEM images a surface pattern of micro-ruggedness typical of nucleation mechanisms and growth. In vitro degradation experiments, carried out for more than 28 weeks, demonstrated that the MNPs delay the scaffold degradation process. Cytotoxicity is appreciated for FeHA content above 20%.

## 1. Introduction

A few years ago, rapid progress in tissue engineering led to the development of certain tissues to replace those damaged either by trauma or disease, or due to congenital defects in sequential order: [1]. Bone regeneration materials are designed to stimulate osteoinductivity, osteoconductivity, osteointegration, biocompatibility, resorbence, and bone-like mechanical properties. Magnetic materials can be introduced into the scaffolds, in order to promote bone formation and cell growth with some of these properties [2]. Magnetic nanoparticles (MNPs) that can be manipulated by an external magnetic field have been used over the past twenty years in both in vivo and in vitro biomedical applications [3]. MNPs should be biocompatible and biodegradable for therapeutic applications. The iron ions in MNPs containing iron and iron oxides, after having metabolized, are added to the iron deposits in the body and are finally incorporated by the erythrocytes or red blood corpuscles into the haemoglobin [4,5]. Some of the therapeutic applications include hyperthermia [6] in which the MNPs transmit heat to concentrate higher temperatures in a specific zone without affecting healthy tissue. Another application is the liberation of pharmaceutical compounds where they can be directed, for example to a tumor, in a selective way, by the application of an external magnetic field [7]. They may likewise be used in non-invasive diagnostic applications such as magnetic resonance imaging (MRI) where the MNPs act as contrast material due to the local modification they produce in the magnetic fields, electric field gradients, and radio waves depending of the type of imaging that is employed.

Some in vitro studies have indicated that magnetic particles have effects on bone marrow-derived mesenchymal stem cells (MSCs), as differentiation, proliferation, intracellular calcium levels, and matrix formation [8,9]. However, one of the problems with MNPs is the strength of the external magnetic field that must be applied to ensure an appropriate time of residence. This problem of timing can be solved by introducing magnetic scaffolds close to the point of medical treatment, with minimally invasive surgery. The scaffolds respond directly to the external magnetic field, thereby circumventing the difficulties of magnetic design guides [9]. With respect to this scope, magnetically active microenvironments have been developed showing increased MC3T3-E1 pre-osteoblast cell proliferation both under magnetic and magnetoelectric stimuli [10]. The latter approach is particularly interesting for bone tissue engineering due to the natural piezoelectric properties of bone [11] and can be achieved when the magnetic fillers are introduced in electroactive polymer-based scaffolds such as the ones based on poly (vinylidene fluoride) (PVDF) or poly (L-lactide) acid (PLLA), [11] among others.

These types of scaffolds should show appropriate long-lasting mechanical properties, hence the use of nanohydroxyapatite (nHA), which has similar properties to the mineral components of calcium hydroxyapatite in bones. Some authors have synthesized iron-doped hydroxyapatite Fe^2+^ and Fe^3+^ [8,9,12,13,14] for medical applications such as magnetic drug carrier, reference [7] and hyperthermia [12], among others. Similarly, magnetic nanospheres have been synthesized from Fe-doped hydroxyapatite [4,13]. A few have synthesized scaffolds with ferrofluids or with magnetite [14]. 

In this paper, the authors report on the development of highly porous magnetic PLLA scaffolds with different concentrations of nanohydroxyapatite doped with iron. The nHA particles are bioactive and biocompatible, and the fact of doping with iron provided them with magnetic properties. The results of an indirect cytotoxicity evaluation of the scaffolds are described using an adaptation of the ISO 10993-5 standard test method. A full characterization of the synthetized nanoparticles and a study of their magnetic behavior and the in vitro degradation of the scaffolds was performed. In vitro results cannot be extrapolated to in vivo but some conclusions offer very valuable information on the in vivo behavior of these devices for tissue engineering.

## 2. Experimental Section

### 2.1. Materials

Poly-l-lactide (PLLA) with a content of residual solvent and residual monomer, in each case of less than 0.01%, was purchased from Biomer L9000 (Biomer, Forst-Kasten, Krailling, Germany). A 1,4 dioxane (Panreac Barcelona, Spain) was used as the solvent. The weight-average relative molecular weight Mw = 141,940, Mn = 95,680 and the polydispersity Mw/Mn = 1.4835 of PLLA were measured with gel permeation chromatography (GPC) (Perkin Elmer 200, Waltham, MA, USA) in tetrahydrofuran (THF). GPC was prepared with a THF solvent using a Perkin Elmer 200 reflective index detector. Calibration adhered to polystyrene standards at a flow rate of 1ml/min. Phosphate-buffered saline (PBS) solution in water purchased from Fluka Analytical (Sigma-Aldrich, Saint Louis, MO, USA), was used as the degradation fluid at a pH of 7.2. The Fe-doped hydroxyapatite (FeHA) nanoparticles were synthesized with FeCl_2_·4H_2_O (98% purity, Sigma-Aldrich, Darmstadt, Germany), FeCl_3_·6H_2_O (97% purity, Sigma-Aldrich, Germany), Ca(OH)_2_ (Honeywell-Fluka, Morristown, NJ, USA), and distilled water (p.a. Panreac, Barcelona, Spain).

### 2.2. Synthesis of Magnetic Nanoparticles

The nanohydroxyapatite precipitate doped with iron was obtained by following the method proposed by Tampieri et al. [12], adding a solution drop by drop of phosphoric acid to a basic suspension of Ca(OH)_2_, containing Fe^2+^ and Fe^3+^ ions, with constant agitation and heating over two hours. The total concentrations of Fe ions as opposed to the Ca ions were adjusted to obtain Fe/Ca = 20 mol %. The reaction products were maintained under stirring and heating for one hour at 60 °C. Subsequently, the precipitate was left for 24 h to age at room temperature with no stirring. The precipitate was separated from the mother solution by means of centrifugation at 5000 rpm and rinsed with distilled water three times. It was then lyophilized, crushed in an agate mortar and, finally, passed through a sieve with a mesh size of 150 µm.

### 2.3. Fabrication of PLLA/FeHA Porous Scaffolds

The protocol for the synthesis of the PLLA/FeHA composite scaffolds previously described in [15] was followed. In short, a PLLA solution was prepared in 1,4 dioxane (2.5% (*w/v*)). Then, a homogenous dispersal of FeHA particles in the above suspension was ensured with ultrasonic stirring. Freezing and freeze-drying weight (LyoQuest of Telstar, Barcelona, Spain) of the resultant solutions for five days yielded highly porous composite scaffolds with different FeHA proportions of total polymer mass (0%, 10%, 30%, 70%, and 80%). This method yielded porous foam scaffolds with porosities of up to 90%.

### 2.4. Cytotoxicity Assay

Membrane sterilization: 0.1 g·mL^−1^ membranes were cut and sterilized under UV for 2 h before cell seeding (1 h each side) for the in vitro assays. Then, each sample was washed five times for five minutes in a phosphate-buffered saline (PBS, Sigma-Aldrich, Saint Louis, MO, USA) solution.

Cytotoxicity assay: an adaptation of the ISO 10993-5 standard test method was used to conduct the indirect cytotoxicity evaluation of the samples. Briefly, the samples were immersed in a 24-well tissue culture polystyrene plate, to prepare the extraction media, with Dulbbecco’s Modified Eagle’s Medium-high glucose (DMEM) containing 1 g·L^−1^ glucose (Sigma-Aldrich, Saint Louis, MO, USA) supplemented with 10% of fetal bovine serum(FBS) (Biochrom, Canbourne, CB, UK) and 1% penicillin/streptomycin P/S (Biochrom, Canbourne, CB, UK). They were immersed at 37 °C in a 95% humidified air containing 5% CO_2_ and incubated for 24 h.

Similarly, the MC3T3-E1 cells were seeded in a 96-well tissue culture polystyrene plate at a density of 3 × 10^4^ cells·mL^−1^ and then incubated for 24 h to allow the cell attachment on the plate. Then, the culture medium was removed from the 96-well tissue culture polystyrene plate and the wells (100 µL) were filled with the as-prepared extraction media (from the samples). Subsequently, the cells were incubated for 72 h and then, the evaluation of the cell viability was quantified with a 3-(4,5-dimethylthiazol-2-yl)-2,5-diphenyltetrazolium bromide (MTT, Sigma-Aldrich, Saint Louis, MO, USA) assay. The cell culture medium was employed as a negative control and 20% dimethylsulfoxide (DMSO, Sigma-Aldrich, Saint Louis, MO, USA) was used as a positive control.

The mitochondrial activity of the cells, which reflects the viable cell number, was measured with the MTT assay. The medium of each well was removed after 72 h, replacing it with the fresh medium containing 10% MTT solution (stock solution of 5 mg MTT·mL^−1^ in PBS). The viable cells with active metabolism converted the MTT into a purple-colored formazan product. Following an incubation period of 2 h, the MTT crystals were dissolved with DMSO and the optical density was measured at 570 nm.

Four replicate samples and controls yielded all the quantitative results and were analyzed as the average of viability ± standard deviation (SD).

Equation (1) was used to calculate the percentage of cell viability.

(1) $$\mathrm{Cell}\;\mathrm{viability}\;\left(\%\right)=\frac{\mathrm{absorbance}\;\mathrm{of}\;\mathrm{sample}}{\mathrm{absorbance}\;\mathrm{of}\;\mathrm{negative}\;\mathrm{control}}$$

### 2.5. In Vitro Degradation

For the degradation experiment, the scaffolds were cut into rectangular pieces and weighed. They were then immersed in glass test tubes containing 10 mL of PBS. The tubes were put into an incubator at 37 °C. The pieces were recovered and wiped after 1, 5, 8, 10, 12, 16, 20, 25, and 28 weeks and then weighed to determine their water absorption. A pH meter PCE 228 (PCE Instruments, Pons, Alicante, España) was used to determine the pH alteration in the medium. The degraded scaffolds were dried over two weeks to a constant weight in a freeze-dryer (LyoQuest of Telstar, Barcelona, Spain).

The following equation was used to calculate the water absorption percentage, Wa%: *

(2) $$\mathrm{Wa}\%=\frac{\mathrm{Ww}-\mathrm{Wr}}{\mathrm{Wr}}\times 100$$

where, Ww was the weight of the wet/swallow sample after removing the surface water. Wr was the residual weight of a dry scaffold after degradation.

Weight loss percentage (WL%) was estimated with the following equation:

(3) $$\mathrm{WL}\%=\frac{\mathrm{Wo}-\mathrm{Wr}}{\mathrm{Wo}}\text{×}100$$

where Wo was the original mass of the scaffold.

### 2.6. Scanning Electron Microscopy (SEM) and Transmission Electron Microscopy (TEM) Analysis

SEM (HITACHI S-3400N, Tokyo, Japan) was used to study the scaffold morphology. A gold coating was applied to the samples, prior to analysis, in a JEL Ion Sputter JFC-1100 ((JEOL, Peabody, MA, USA) at 1200 V and 5 mA, to avoid sample charging under the electron beam.

A PHILIPS CM120 Biofiller with a STEM module and elemental mapping and filtering of images with electron energy-loss spectroscopy (EELS) was used to perform the Transmission Electron Microscopy (Philips Ibérica, Madrid, España).

### 2.7. X-ray Diffraction Analysis

An Xpert diffractometer PANalytical PRO (Philips, Almelo, Netherlands) equipped with a copper tube (λCuKα media = 1.5418 Å, λCuKα 1 = 1.54060 Å, and λCuKα 2 = 1.54439 Å), a vertical goniometer (Bragg-Brentano geometry) a programmable divergence slit, an automatic sampler, a secondary graphite monochromator, and a PIXcel detector (Philips, Almelo, Netherlands)) was used to perform X-ray diffraction (XRD) analysis.

### 2.8. Magnetic Analysis

Magnetic measurements were performed at physiological temperature (310 K) on a Vibrating Sample Magnetometer (VSM). The magnetometer, developed at the University of the Basque Country, was calibrated with pure (99.995%) nickel. The available magnetic field range was ±1.8 Tesla (18 kG), and it had a resolution of ±20 µTesla (0.2 G), and a moment sensitivity of 10^−8^ Am^2^ (10^−5^ emu). The data were normalized to the sample mass, determined by an analytical balance calibrated to ±0.05 mg. In all cases, the overall accuracy of the magnetic saturation values was over 1%.

### 2.9. Differential Scanning Calorimetry (DSC)

A TA Instruments calorimeter at a range of 4–120 °C was used to perform the DSC measurements. After heating the samples at 5 °C/min (first run), cooling them at 10 °C/min (second run), and finally heating them at 5 °C/min (third run), the degree of crystallinity, Xc%, of the samples was evaluated with the following equation:

Xc% = 100 (∆Hm1/∆H°m)
(4)

where ∆Hm1 is the enthalpy of melting measured in the first run and ∆H°m is the enthalpy of melting of a totally crystalline PLLA (∆H°m = 93 J/g) [16]. The crystallizable fraction (CF%) of the samples was calculated with the following equation [8]:

CF% = 100 (∆Hc/∆Hm1)
(5)

### 2.10. Fourier-Transform Infrared (FTIR)Spectroscopy

Infrared spectra of the samples were recorded on a Thermonicolet Avatar 370 Fourier-transform infrared (FTIR, Thermo Electron Corporation, Waltham, MA, USA) spectrophotometer equipped with an attenuated total reflectance attachment with ZnSe crystal. Spectra were repeatedly scanned 32 times between 4000 and 650 cm^−1^ at a resolution of 4 cm^−1^.

## 3. Results and Discussion

### 3.1. Characterization of FeHA

**Figure 1:**
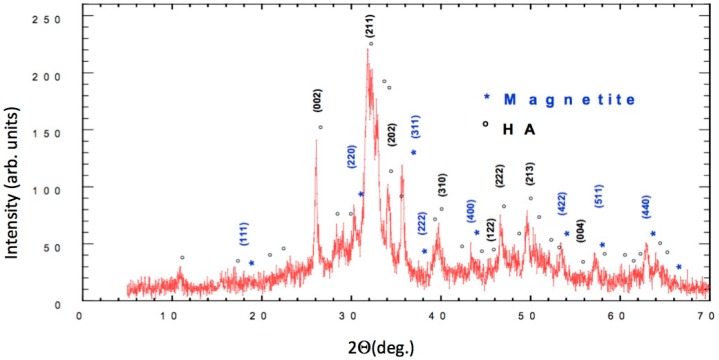
Cu Kα diffractogram of the synthetized nano-Hydroxyapatite (nHA), (*) Magnetite reflections, (**◦**) nHA reflections.

The results of X-ray powder diffraction (XRD) are detailed in Figure 1, in which both the sites and the intensities of the diffraction peaks are shown. The circled peaks representing Ca_5_(PO_4_)_3_(OH) are consistent with the standard pattern for JCPDS Card No. 25–166.

The characteristic peaks for JCPDS Card No. 19–629 indicated a very high content of magnetite (marked with an asterisk in Figure 1 in the synthesized FeHA, which might have been due to the high temperature of the synthesis (60 °C). The results were quite different from the ones presented by Panseri et al. because of the very high content of magnetite [2].

Structural analysis provided evidence of the substitution of ions Fe (Fe^2+^ and Fe^3+^) in the HA structure. The crystallite size (D) at around 30 nm was determined using Scherrer’s formula [17]. FULLPROF [18] software was used to calculate the full width and half of the maximum intensity.

**Figure 2:**
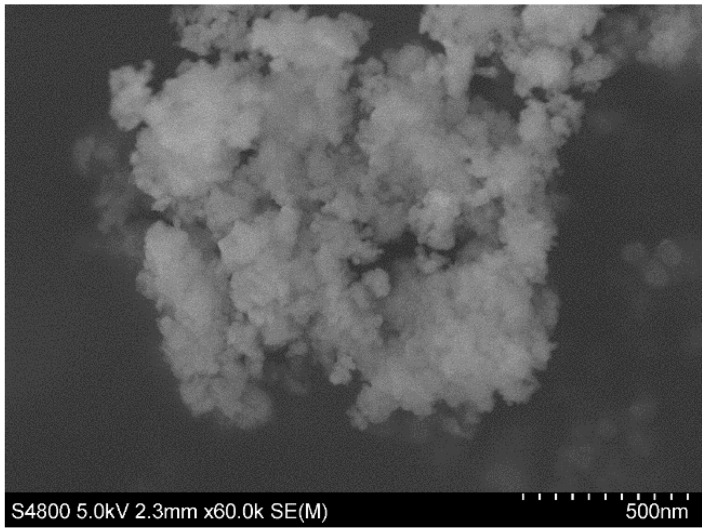
Scanning electron microscopy (SEM) micrographs of Fe-doped hydroxyapatite (FeHA) nanoparticles.

**Figure 3:**
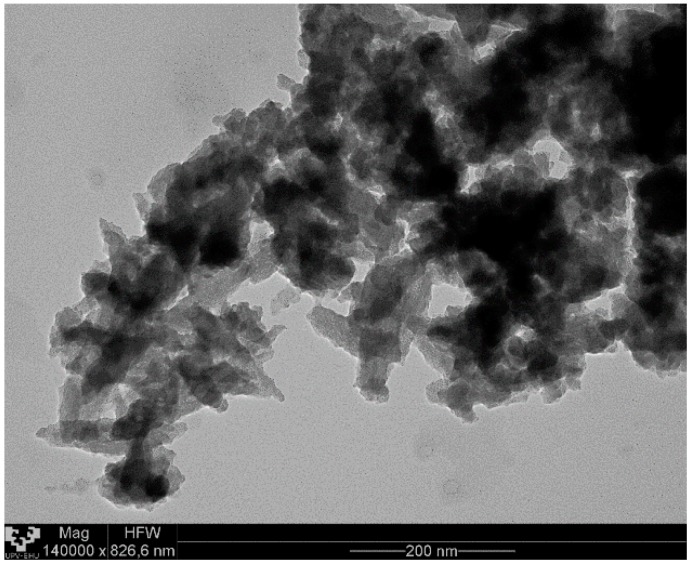
Transmission electron microscopy (TEM) micrographs of FeHA nanoparticles.

The SEM and TEM analysis of the FeHA nanoparticles’ morphology showed a large number of dark spots corresponding to inclusions of iron-rich phases and quite heterogeneously sized particles of approximately 25 nm in width and 100–150 nm in length (see Figure 2 and Figure 3, respectively).

### 3.2. Magnetic Properties

**Figure 4:**
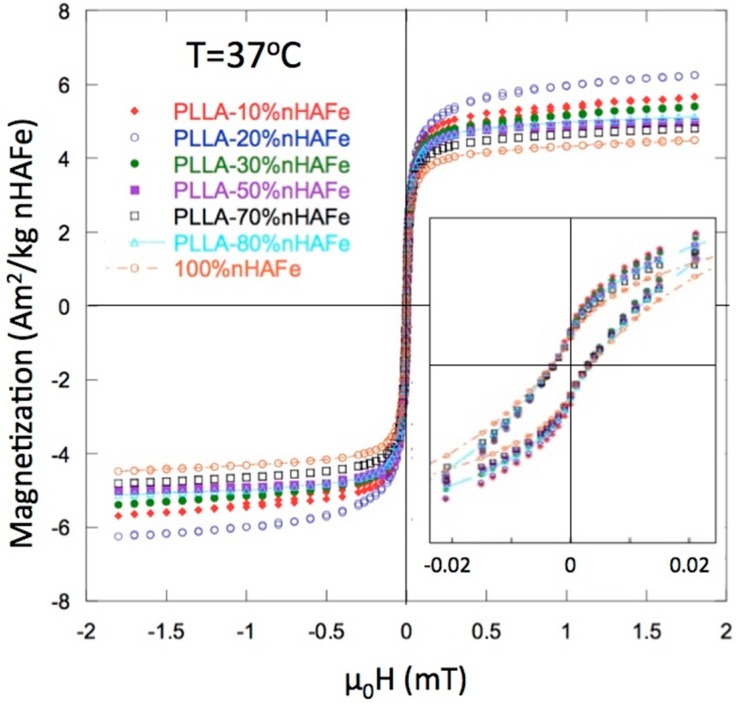
Magnetization curves for FeHA and poly (L-lactide) (PLLA)-FeHA scaffolds. The inset shows the close-up of the “wasp-waist” loops described in the text.

Figure 4 shows the hysteresis loops of the PLLA/FeHA scaffolds at 37 °C, as measured in a Vibrating Sample Magnetometer (VSM). The main magnetic parameters, saturation magnetization (Ms) and coercive field (Hc), are listed in Table 1. The true content of FeHA is included, calculated from the experimental value of the magnetization. The coercivity of the initial FeHA and of all the composite scaffolds was quite similar: µoHc ≈ 2–3 mT. The lowest coercivity level was observed in the PLLA/FeHA 80 wt % scaffold. This value, although it was low for the largest nanoparticle concentrations, was not indicative of superparamagnetic (SPM) behavior [12]. The ferromagnetic (FM) character of the nanoparticles was also clear from the rapid saturation of the magnetization.

The “wasp-waist”-shaped loops, shown in the inset of Figure 2, indicate a combination of low Hc and high Hc compounds, in the two magnetic nanoparticle phases. The low Hc phase was linked to FeHA and the high Hc phase was linked to a segregation of some magnetite (Fe_3_O_4_) during the preparation of the FeHA particles.

### 3.3. SEM

**Figure 5:**
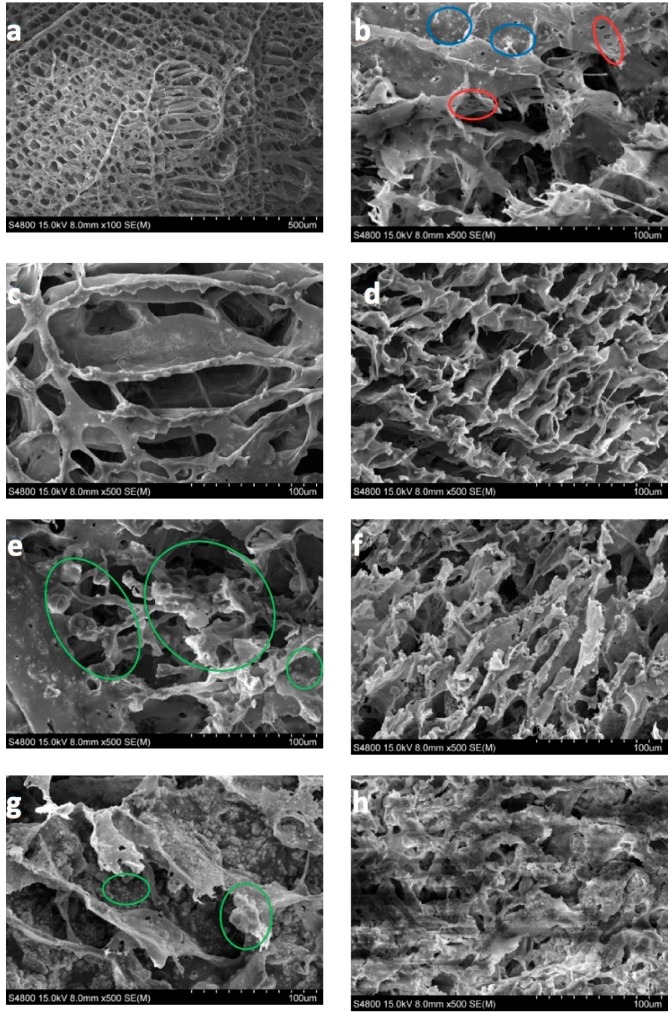
100 × magnification SEM micrographs of: (**a**) PLLA as prepared, (**b**) PLLA-30% FeHA after 8 weeks degradation, (**c**) PLLA-10% FeHA as prepared, (**d**) PLLA-10% FeHA after 25 weeks degradation, (**e**) PLLA-30% FeHA as prepared, (**f**) PLLA-30% FeHA after 25 weeks degradation, (**g**) PLLA-70% FeHA as prepared, (**h**) PLLA-70% FeHA after 25 weeks degradation.

In this section, the morphology of the magnetic scaffolds manufactured with the thermally induced phase separation (TIPS) technique, the influence of magnetic nanoparticle concentrations, and the in vitro degradation process are all studied. It may be seen, in Figure 5, that the scaffolds with a lower number of nanoparticles present an anisotropic tubular morphology with a ladder-like internal structure and pore sizes of 25–250 µm, characteristic of a solid–liquid phase separation (see Figure 5a,c,d) [19]. The larger amounts of FeHA scaffold pores were of a smaller size (approximately 15–180 µm), and presented a much more isotropic morphology, due to the perturbation produced by the nanoparticles in the crystallization of the solvent (see Figure 5e,g). In fact, the walls of the pores contain hill-type structures, with small peaks (green circles) that gave the walls a micro-roughness pattern, typical of nucleation and growth that correspond to liquid–liquid phase separation [20,21,22].

As the process of degradation advanced, changes in scaffold morphology may be appreciated, showing a change from a smooth to a rougher appearance, with micro-porosities appearing on the scaffold walls (red circles) and particle coalescence on the walls of the porous carrier (see Figure 5b) (blue circles). This might be due to the degradation that gradually deposits a layer of FeHA from the surface of the scaffold into the internal pores.

### 3.4. Thermal Analysis

The DSC data are shown in Table 2. The values of Tm = 184 °C, Tc = 56 °C, and Tg = 96 °C remained constant during in vitro degradation, in PBS, at pH 7.2, over 25 weeks. Some authors have reported decreasing of Tc and Tg after 12 months for scaffolds without nanoparticles [20]. ∆H_c_, ∆H_m_, and Xc% decreased with degradation time, although some samples (10 wt % FeHA and 30 wt % FeHA) slightly increased crystallinity around the 12th week of degradation. This increase, discussed in the literature [15,23,24,25,26,27], is attributed to the splitting of the chains, which as they shorten, reorder more easily and form crystals.

The crystallinity of the polymer composites, as shown in Table 2, decreased during the degradation process, unlike what the pure polymer does. The crystalline fraction (CF) simultaneously increased in almost inverse proportionality with the crystallinity, that is, the lower the polymer crystallinity, the higher the CF of the samples. This is a behavior that might be related to the polymeric chain length. The samples with the lowest crystallinity had shorter polymeric chains where they were affected by degradation and therefore showed a loss of order (i.e., lower crystallinity); in the second run, these shortened polymeric chains, due to their reduced molecular weight, were able to crystallize, with a consequent increase in CF. It may, therefore, be affirmed that the samples with the lowest polymer crystallinity and the highest CF correspond to the lowest polymeric chain length [28].

### 3.5. Water Uptake

**Figure 6:**
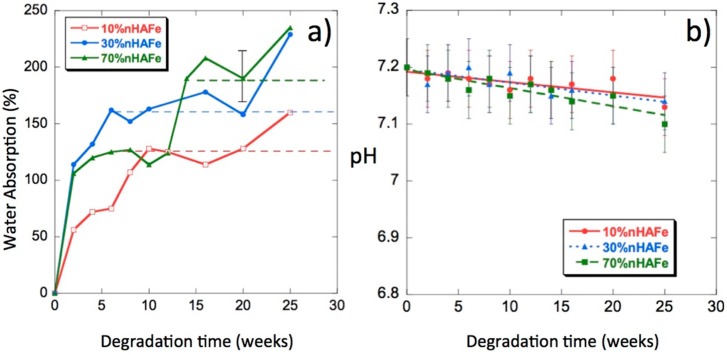
(**a**) Water absorption by PLLA/FeHA scaffolds vs. degradation time; (**b**) pH of the phosphate-buffered saline (PBS) solution vs. degradation time.

Figure 6a shows the water absorption percentages as a function of degradation time. We can see from these curves that the introduction of bioactive magnetic nanoparticles in the scaffolds increased their capacity to absorb PBS; some samples reached absorption percentages of approximately 250% at 25 weeks of in vitro degradation. In a previous paper on PLLA/nHA [29], the highest absorption (approximately 200%) occurred in the polymer without nHA and the addition of nanoparticles decreased the absorption levels. The doping of nHA with Fe^2+^ and Fe^3+^ in the form of magnetite increased water absorption. Such a behavior was expected in a composite polymer material that is hydrophobic, whereas FeHA is very hydrophilic. Water is a degradation medium and its absorption levels are determined by the balance between the oligomer dissolution rate and the water uptake of residual material in the solution. The rate of polymer hydrolysis was dependent on the amount of water hydrogen bound to the oxygen in the ester carbonyl groups [29,30,31]. The absorption of large amounts of water will not mean an increase in the degradation rate.

### 3.6. pH

The release of acidic products, due to hydrolysis, gives an idea of the progress of the degradation process [32]. In Figure 6b, we can see the pH changes of the PLLA/FeHA scaffolds as a function of degradation time. The pH values decreased very slowly up until the 25th week of the degradation process. The highest variations in pH were experienced by the composition of the highest nanoparticle contents (PLLA/FeHA 70 wt %), results that were coincident with the higher water absorption and weight loss.

### 3.7. Mass and Weight Loss

The first consequence of the degradation was the decrease of molecular weight, followed by the reduction of the tensile strength and, finally, the decrease of mass. Table 3 presents the changes in the weight-average relative molecular weight, Mw, Mn, and polydispersity I = Mw/Mn against degradation time. The Mw of the PLLA used to manufacture the scaffolds must be higher than 50,000 g/moL, for its application in bone fixation devices to be feasible [33].

While the mass remained unchanged during degradation, the decrease in molecular weight was clear (see Table 3). The decreases, however, were not very large due to the high crystallinity of the scaffolds. This behavior can be explained by the aqueous solution penetrating the polymer and initiating the process of hydrolytic degradation during the first stage of hydrolysis, mainly in the amorphous regions. At this stage, a rapid decrease in molecular weight was observed, but with no significant loss of mass, because the broken molecules were still not soluble in water and remained inside the sample. In the second step, the mass loss increased, and the formation of a lactic acid monomer was observed with a slight change in molecular weight. At this point, the long polymer chains were converted into shorter water-soluble fragments that dissolved in the medium. PLLA solubilizes in water at Mn ≤ 20,000 [27] during the 25th week of degradation. Looking carefully at Table 3, one might think that the scaffolds are at the end of the first stage of the degradation process. For compositions of PLLA/FeHA 30 wt % and 70 wt % after 25 weeks of degradation, a second peak appeared in the GPC with a longer retention time, corresponding to smaller chains. At the end of the degradation process, a decrease in the values of Mw and Mn of approximately 50% was observed for the samples with the lowest percentage of nanoparticles.

**Figure 7:**
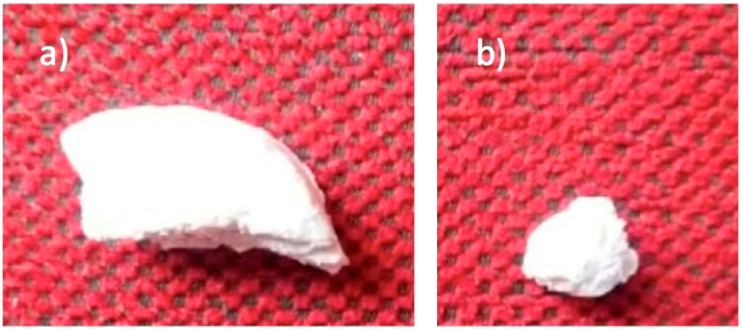
Morphology of PLLA/FeHA 10 wt % (**a**) before and (**b**) after in vitro degradation.

In Figure 7, the change in size and morphology of the PLLA/FeHA 10 wt % sample can be observed before and after the in vitro degradation process. Although mass remained invariable, the same could not be said of molecular weight. The chains split to form smaller ones, because the macromolecular chains could be trapped in the crystalline regions, due to the high crystallinity of the polymer.

### 3.8. FTIR

**Figure 8:**
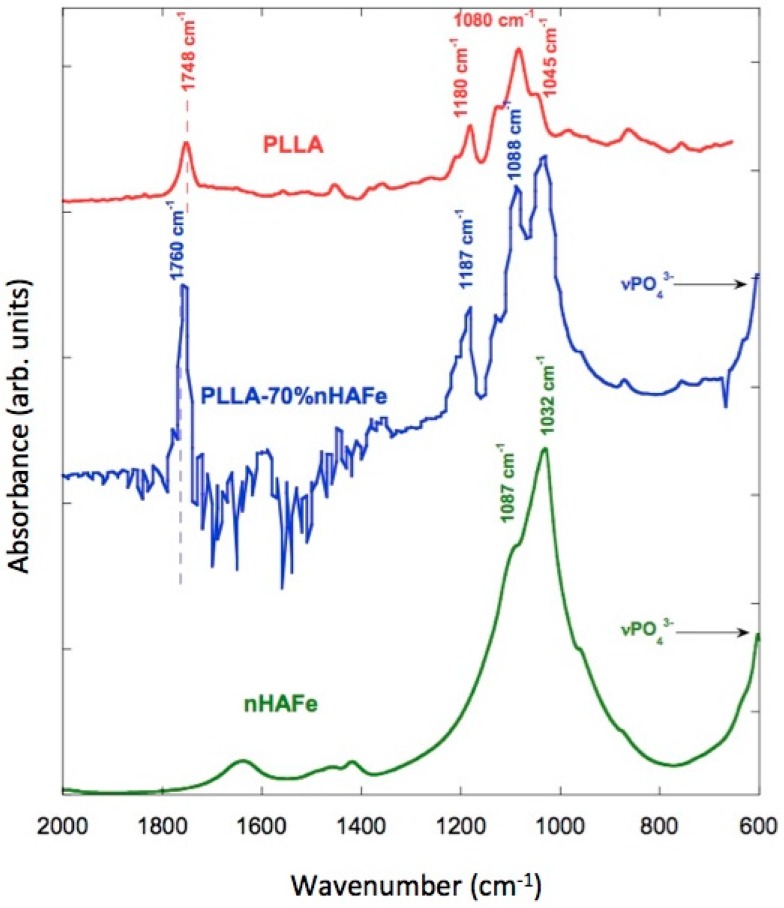
Fourier-transform infrared spectroscopy (FTIR) spectra of FeHA, PLLA/FeHA 70 wt % and pure PLLA.

The IR spectra of the scaffolds were recorded with Fourier-transform infrared (FTIR) spectroscopy and an attenuated total reflectance (ATR) accessory, so as not to modify the samples. FeHA, PLLA, and PLLA/FeHA 70 wt % absorption spectra can be observed in Figure 8. It shows how some bands vary slightly in position, which may be indicative of interactions between the calcium and iron cations of the nanoparticles and the carbonile polymer groups. These bands are: stretching C=O displaced from 1748 cm^−1^ to 1760 cm^−1^, stretching C–O displaced from 1180 cm^−1^ to 1187 cm^−1^ and from 1080 cm^−1^ to 1088 cm^−1^.

The peak at 1045 cm^−1^, present in the pure polymer, overlapped with the ν_3_PO_4_^3−^ band located between 1087–1032 cm^−1^ of the nanohydroxyapatite. It is worth mentioning the appearance of inverted bands in the PLLA/FeHA 70 wt % composite spectra. This anomalous form of IR band is due to the “Christiansen effect” [34].

In Figure 8, the vibration modes of the PO_4_^3−^ group in relation to the absorption of PLLA/FeHA 70 wt % were ν_1_, ν_2_ and ν_4_, whereas mode ν_1_ was present at 962 cm^−1^, mode ν_3_ appeared at 1087 and 1032 cm^−1^, and the band at 603 cm^−1^ was attributed to vibration mode ν_4_. A double peak was observed at 1457 and 1420 cm^−1^, attributed to the ν_3_ CO_3_^2−^ vibration mode, which is a carbonate ion type B deficient in Ca [35].

**Figure 9:**
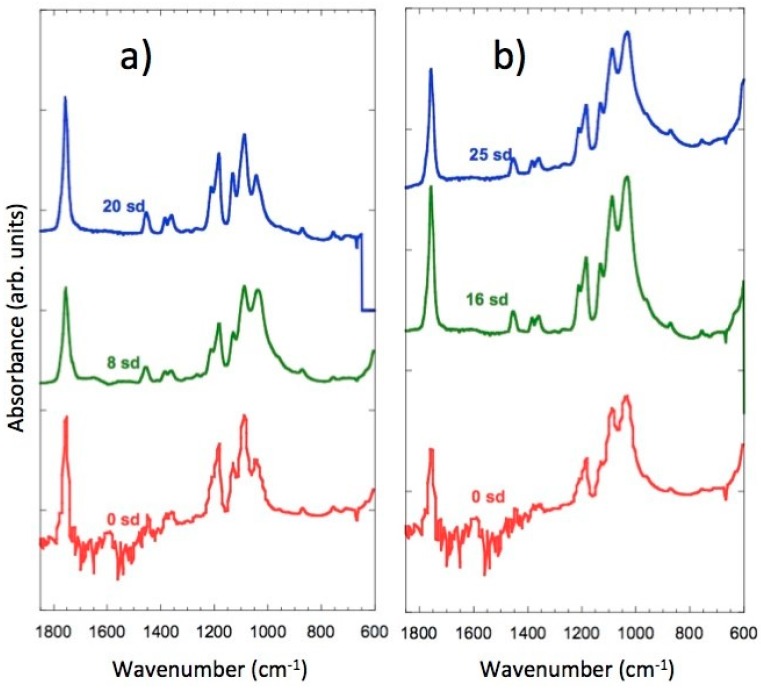
FTIR spectra of: (**a**) FeHA, PLLA/FeHA 30 wt % as prepared and after 8 and 20 weeks degradation. (**b**) FeHA, PLLA/FeHA 70 wt % as prepared and after 16 and 25 weeks degradation.

In Figure 9a,b, the spectra of the samples of PLLA/FeHA 30 wt % and 70 wt % during the degradation process can be observed. There are no appreciable changes, as might be expected, in the C=O band at 1760 cm^−1^, because of hydrolysis-induced splitting that caused bond breakage, due to the saturation of the band. However, in their analysis of the hydrolytic degradation of aliphatic polyesters by FTIR, Partini et al. focused their study on the band at 1570 cm^−1^, corresponding to the carboxylic end groups. In this study, the 1570 cm^−1^ band was not appreciable for the polyester prior to degradation, although it started to appear during degradation [35]. The appearance of the carbonyl end band was not observed in any of the compositions, so there was no evidence of degradation in this system during the 25 weeks of in vitro degradation.

### 3.9. Cytotoxicity

**Figure 10:**
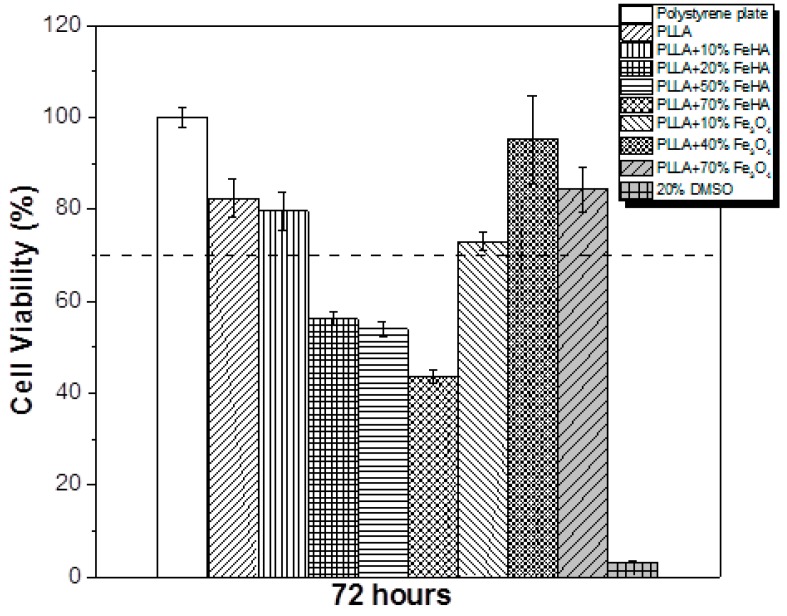
Cell viability of MC3T3-E1 pre-osteoblast cells in contact with the as-prepared extraction media exposed with the different samples up to 72 h.

The tissue engineering potential of the samples in biomedical applications was evaluated by studying the cytotoxicity of the PLLA-based composites with different concentration of Fe_3_O_4_ and FeHA. The effect of the polymer extract medium on the metabolic activity of the MC3T3-E1 cells was performed using the MTT assay, and the results after 72 h are presented in Figure 10.

**Figure 11:**
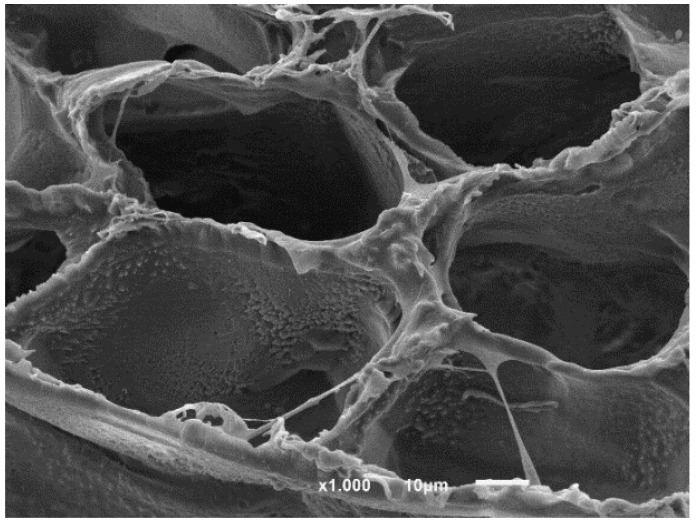
Cell morphology of MC3T3_E1 pre-osteoblastos seeded on PLLA.

Neat PLLA has previously been reported as biocompatible and not cytotoxic [36]. According to the ISO standard 10993-5, materials are considered cytotoxic when the cells suffer a viability reduction larger than 30%, which occurred in the study’s PLLA samples with 20%, 50% and 70% FeHA. The samples containing Fe_3_O_4_ were not cytotoxic, in agreement with the studies of other researchers [37]. In relation to the samples with FeHA, and because both PLLA and hydroxyapatite [38] are not cytotoxic, some of the synthesized products used for the preparation of the samples may have remained encapsulated within the materials or in the interfacial regions, leading to cytotoxicity for concentrations above 20% FeHA. Figure 11 shows the cell morphology of MC3T3-E1 pre-osteoblastos seeded on PLLA after three days.

## 4. Conclusions

In this study, novel PLLA magnetic nanohydroxyapatite scaffolds were prepared and fully characterized. The ferromagnetic behavior of the scaffolds was confirmed by the rapid saturation and appreciable coercivity of the magnetization curves of the magnetic particles, approximately 30 nm in size, intermeshed in the scaffolds. The study of the scaffold morphologies identifies a liquid–liquid separation system in the fabrication process with proportions of magnetic nanoparticles of over 20%. Although changes in molecular weight were observed during in vitro degradation, almost no mass loss was observed, due to the entrapment of the macromolecular chains in the crystalline regions and the high crystallinity of the polymer. The DSC results of percentage crystallinity, Xc%, have presumably diminished MNP crystallinity, thereby reducing the mobility of the chains and leaving some synthesized products encapsulated in the structure of the scaffolds.

## Figures and Tables

**Table 1:** Nominal FeHA content (wt %), Ms = Magnetization at 1.5 T, in the saturated region (Am^2^/kg of FeHA), µ0Hc = coercivity (mT), and FeHA content recalculated from the measured magnetization.

% FeHA Nominal	Moment (Am^2^/kg _FeHA_)	µ_0_ H_c_ (mT)	% FeHA Recalculated
100 ^(1)^	4.45	3.18	-
10	5.58	2.77	12.5
20	6.16	2.73	27.6
30	5.33	2.64	36
50	4.86	2.65	54.5
70	4.77	2.50	74.9
80	4.86	2.27	87.2

^(1)^ Pure FeHA powder taken as reference.

**Table 2:** Parameters obtained by thermal analysis on the PLLA-FeHA system: dt (weeks) = degradation time in weeks, Tm = melting point (°C), ∆Hm = melting enthalpy (kJ/kg), Tg = glass transition temperature (°C), Tc = crystallization temperature (°C), ∆Hc = crystallization enthalpy (kJ/kg), Xc = crystalline fraction (%), calculated as Xc% = 100 [(∆Hm1 − ∆Hcc)/∆Hm_0_] with ∆Hm_0_ = 93 J/g [16], CC = crystallization capacity (%), calculated as: CF % = 100(∆Hc/∆Hm1).

PLLA/FeHA% dt (Week)	First Run				Second Run			X_c_%	CF%
	Tm (°C)	ΔHm (J/g)	Tcc (°C)	ΔHcc (J/g)	Tg (°C)	Tc (°C)	ΔHc (J/g)		
**0%**	184	41.1	76	4.7	56	96	2.3	39	6
**10%**	183	44.1	75.5	3.4	58.5	98	9.7	44	22
**4 dt**	183	41.8	76.5	2.3	58	97	8.8	42	21
**8 dt**	182	43.2	75	1.9	59	97	6.9	44	16
**12 dt**	182	44	-	-	58	98	6.8	47	15
**16 dt**	182	40.7	-	-	57	99	6.4	44	16
**20 dt**	182	40.1	77.5	1.5	58	97	5.7	42	14
**25 dt**	181	38	77	2.7	56	98	5.2	38	14
**20%**	184	36.1	76.1	4.9	60	101	18.2	33.5	50
**30%**	183	31.7	-	-	61	103	20.4	34	64
**4 dt**	183	24.2	77	2.5	62	105	19	23	78
**8 dt**	182	28.8	77	1.43	61	102	17	29	59
**12 dt**	182	29	86	1.3	61	103	15	30	52
**16 dt**	181	30.3	-	-	58	100	12	33	40
**20 dt**	181	28.6	74	2.3	57	98.5	10	28	35

**Table 3:** Molecular weight: weight average (Mw), number average (Mn), and polydispersity index (I) of the PLLA/FeHA system.

Sample	Degradation Time (Weeks)	Mw	Mn	I
PLLA	0	144,221	104,042	1.386
PLLA/FeHA 10 wt %	0	98,633	55,206	1.787
	16	94,867	53,228	1.782
	20	92,094	53,123	1.734
	25	52,704	32,946	1.600
PLLA/FeHA 30 wt %	0	66,625	47,961	1.389
	5	55,750	30,984	1.799
	16	49,382	20,093	2.458
	20	89,927	45,269	1.986
	25	49,972	36,484	1.370
	(*)	10,668	9,873	1.081
PLLA/FeHA 70 wt %	0	81,855	44,573	1.836
	15	98,065	45,623	2.149
	25	86,760	53,289	1.628
	(*)	10,982	10,511	1.045
